# Enterovirus 71 induces apoptosis of SH-SY5Y human neuroblastoma cells through stimulation of endogenous microRNA let-7b expression

**DOI:** 10.3892/mmr.2015.3482

**Published:** 2015-03-13

**Authors:** XILING DU, HAIPENG WANG, FUHUI XU, YONGYI HUANG, ZHIXUE LIU, TE LIU

**Affiliations:** 1 School of Life Science and Technology, Tongji University, Shanghai 200092, P.R. China; 2 Shanghai Geriatric Institute of Chinese Medicine, Longhua Hospital, Shanghai University of Traditional Chinese Medicine, Shanghai 200031, P.R. China

**Keywords:** HFMD, enterovirus 71, microRNA, let-7b, cyclin D1, apoptosis

## Abstract

Enterovirus 71 (EV71) is a pathogenic microorganism that causes hand, foot and mouth disease. However, the epigenetic mechanisms behind how EV71 regulates host cell proliferation and apoptosis are unclear. In the present study, the ability of EV71 to induce apoptosis was analyzed in the SH-SY5Y human neuroblastoma cell line and the effect of this virus on the mRNA expression levels of various apoptotic markers, miRNA let-7b and cyclin D1 (CCND1), was also investigated. The results demonstrated that EV71 induced SH-SY5Y cell apoptosis. An MTT assay revealed a significant inhibitory effect of EV71 on cell proliferation between 12–72 h post injection, compared with the control group. Furthermore, quantitative polymerase chain reaction and western blot analyses demonstrated that expression level of the apoptosis inhibitor Bcl-2 was markedly reduced, but the expression levels of the apoptosis-promoting factors Bax, caspase-7, caspase-3 and active caspase-3 were markedly higher in the SH-SY5Y cells 12–48 h after EV71 infection, compared with the non-infected cells. In addition, flow cytometric assays revealed that EV71 arrested the cell cycle of host SH-SY5Y cells. Northern blot analysis revealed a marked miRNA let-7b hybridization signal in the EV71 virus-infected group compared with the non-infected group. Furthermore, western blotting confirmed that the CCND1 protein expression levels were significantly reduced in EV71-infected SH-SY5Y cells. EV71-inhibited SH-SY5Y proliferation was abrogated using let-7b specific 2′-O-Methyl-RNA, which inhibited endogenous miRNA let-7b expression. Thus, EV71 regulated the host SH-SY5Y cell cycle and cell proliferation via stimulating endogenous miRNA let-7b and directly targeting CCND1, therefore EV71 is a potential candidate for antiviral therapy.

## Introduction

More than 500,000 hand, foot and mouth disease (HFMD) cases caused by human enterovirus 71 (EV71) are reported in the People’s Republic of China annually, including 176 fatal cases since March 2008 (1). One study has shown that EV71 and Coxsackie virus A16 (CVA16) are the two predominant causative agents of HFMD, accounting for >70% of recent outbreaks (2). EV71 infection is more frequently associated with serious neurological diseases, such as aseptic meningitis, encephalitis and acute flaccid paralysis, while CVA16-associated HFMD has a milder outcome (1). Although the pathogens that cause HFMD have been confirmed, the association between host cells and the HFMD viruses, and the mechanism by which the HFMD virus induces host cell apoptosis remains unclear. An increasing number of studies have revealed that numerous viral infections, including hepatitis B virus, hepatitis C virus, human immunodeficiency virus and sarcoma-associated herpes virus, among others, are closely associated with the regulation of miRNAs (miRNAs) (3–6). miRNAs are a class of naturally occurring single-stranded short 21–23 nt non-coding RNAs (7,8) that exist in a wide range of eukaryotic organisms (7–12). Each mammalian miRNA prevents the translation of a number of downstream target mRNAs and ultimately results in the inhibition of target gene expression (13,14). Therefore, a shift away from the manipulation of crucial target genes towards miRNA interference techniques may improve the effectiveness of current gene-based diagnostic and therapeutic strategies (9). Although the majority of miRNA studies focus on the growth and differentiation of stem cells (15–17), tumorigenesis (18,19) and other pathological processes (13,14), few studies have focused on the role of miRNAs in the interaction between EV71 and human neurons. Thus far, certain studies have reported that miRNAs are involved in the host response to EV71 infection. Cui *et al* (1) used a deep sequencing approach to determine that 64 miRNAs in host cells exhibited >2-fold expression level changes in response to EV71 infection. Wen *et al* (20) found that miRNA-23b in host cells inhibited EV71 replication through downregulation of the EV71 viral capsid protein (VPl). Zheng *et al* (21) showed that miRNA-296-5p suppressed EV71 replication in host cells by inhibiting two potential targets (2,115-2,135 nt and 2,896-2,920 nt) located in the EV71 genome. Furthermore, Li *et al* (22) demonstrated that the members of the miRNA-548 family, including miR-548b-5p, miR-548c-5p, miR-548i, miR-548j and miR-548n, downregulate the host antiviral response during EV71 or vesicular stomatitis virus infection via direct targeting of interferon-λ1. In addition, Cui *et al* (2) compared host serum miRNA levels in patients with HFMD caused by EV71 and CA16 as well as in healthy individuals. In the sera of patients with the enteroviral infections, 102 miRNAs were upregulated and 26 miRNAs were downregulated. Therefore, altered circulating miRNA profiles have been observed in patients with microbial infections. These results enhance the understanding of miRNA involvement resulting from EV71 infection in HFMD and offer insight into potential prevention and treatment approaches.

Let-7 is a well-known miRNA known to regulate cell cycle and development, that is underexpressed in various types of cancer (23). Restoration of normal let-7 expression levels has been demonstrated to inhibit cancer growth by targeting various oncogenes and inhibiting the key regulators of several mitogenic signaling pathways (23–26). Yu *et al* (26) found that let-7 suppressed self-renewal and tumorigenicity in breast cancer cells by reducing H-RAS and high-mobility group AT-hook (HMGA) 2 expression levels. Furthermore, Schultz *et al* (24) reported that let-7b, a member of the let-7 miRNA family, interfered with the proliferation and growth of primary malignant melanoma cells by targeting and suppressing important cell cycle molecules, such as cyclin D (CCND1). In addition, Dangi-Garimella *et al* (25) revealed that elevated let-7 expression levels inhibited HMGA2 expression and suppressed metastasis in breast cancer cells. In view of this evidence, whether EV71 stimulates endogenous miRNA let-7 expression to inhibit growth and proliferation, and induce apoptosis in host cells was investigated in the present study.

## Materials and methods

### Cell culture and viral infection

The SH-SY5Y human neuroblastoma cell line, which was purchased from the Cell Resources Center of Shanghai Institute of Life Science, Chinese Academy of Sciences (Shanghai, China) was grown in Dulbecco’s modified Eagle’s medium (DMEM) supplemented with 10% fetal bovine serum (FBS), penicillin (100 U/ml), streptomycin (100 U/ml) and 2 mM L-glutamine (all purchased from Hyclone, Logan, USA). The SH-SY5Y cells were at 37°C in a humidified atmosphere of air containing 5% CO_2_. The prototype EV71 was donated by Dr Weihao Li (Handan Municipal Center for Disease Prevention and Control, Hubei, China). The SH-SY5Y cells were infected with EV71 virus as previously described (1,27). Briefly, SH-SY5Y cells were grown to 80% confluence prior to infection. For virus absorption, the cells were infected for 60 min with EV71 at a multiplicity of infection (MOI) of 1, 50% tissue culture infectious doses, in serum-free medium. Following infection, the cells were washed with phosphate-buffered saline (PBS) and maintained at 37°C in DMEM medium with 2% FBS.

### Reverse transcription (RT) and quantitative polymerase chain reaction (qPCR) analysis

Total RNA was isolated from each cell type using TRIzol^®^ Reagent (Invitrogen Life Technologies, Carlsbad, CA, USA) according to the manufacturer’s instructions. The RNA samples were subsequently treated with DNase I (Sigma-Aldrich, St. Louis, MO, USA), quantified and reverse-transcribed to cDNA using the ReverTra Ace-α First Strand cDNA Synthesis kit (Toyobo Co., Ltd., Osaka, Japan). The qPCR was conducted using a RealPlex4 real-time PCR detection system (Eppendorf, Hamburg, Germany) with SYBR Green Realtime PCR Master mix (Toyobo Co., Ltd.). The qPCR amplification was performed over 40 cycles of denaturation at 95°C for 15 sec and annealing at 58°C for 45 sec, and target cDNA was measured using the relative quantification method. The comparative threshold cycle (Ct) calculation was used to determine the relative gene expression levels, normalized to 18S rRNA. For each sample, the Ct values were normalized using the formula: ΔCt = Ct_genes - Ct_18S RNA. The relative expression levels were calculated using the formula: ΔΔCt = ΔCt_all_groups - ΔCt_blank control_group. The values used to plot relative gene expression levels were calculated using 2^−ΔΔCt^. The primers used for the cDNA amplification were as previously described (15).

### Transmission electron microscopy (TEM) analysis

TEM analysis was conducted as previously described (28). Briefly, each group of cells was fixed in 1% glutaraldehyde 1 h prior to post-fixing in 1% osmium tetroxide for 1 h, then the cells were dehydrated in an acetone dilution series and embedded in resin 12 (Ted Pella, Inc., Redding, CA, USA). Transverse sections (900 nm) were stained with toluidine blue (Sigma-Aldrich, St. Louis, MO, USA) and examined using a Nikon Eclipse 80i microscope (Nikon Instruments, Inc., Melville, NY, USA). Ultra-thin sections (70 nm) were stained with 1% uranyl acetate and 1% lead citrate, and examined using a JEM-1230 (JEOL, Tokyo, Japan) transmission electron microscope.

### Terminal deoxynucleotidyl-transferase-mediated dUTP nick end labeling (TUNEL) assay

TUNEL assay was performed as previously described (27,29). Briefly, each group of cells treated as indicated was fixed with 4% paraformaldehyde, rinsed with PBS, then permeabilized with 0.1% Triton X-100 for fluorescein isothiocyanate (FITC)-end-labeling the fragmented DNA of apoptotic SH-SY5Y cells using a TUNEL cell apoptosis detection kit (Beyotime Institute of Biotechnology, Shanghai, China). The FITC-labeled TUNEL-positive cells were imaged under a fluorescent microscope (DMI3000; Leica, Allendale, NJ, USA) using 488 nm excitation and 530 nm emission.

### Northern blotting

Northern blotting was conducted as previously described (17). For all cell treatment groups, total RNA was isolated from each cell type using TRIzol reagent (Invitrogen Life Technologies), according to the manufacturer’s instructions. The RNA samples were subsequently treated with DNase I (Sigma-Aldrich), and 20 *μ*g total RNA was analyzed on a 7.5 M urea, 12% polyacrylamide denaturing gel, then transferred to a Hybond N+ nylon membrane (Amersham, Freiburg, Germany). The membranes were cross-linked using ultraviolet light for 30 s at 1,200 mJ/cm^2^ and hybridized to the let-7b antisense starfire probe (GenScript, Piscataway, NJ, USA), for the detection of 21 nt let-7b fragments, according to the manufacturer’s instructions. Following washing, the membranes were exposed to Kodak XAR-5 film for 20–40 h (Sigma-Aldrich Chemie GmbH, Steinheim, Germany). A human U6 snRNA probe, 5′-G CAGGGGCCATGCTAATCTTCTCTGTATCG-3′, served as a positive control. The exposure time was 15–30 min.

### Western blotting

Total proteins extracts of each cell treatment group were resolved by 12% SDS-PAGE and transferred onto polyvinylidene difluoride (Millipore, Billerica, MA, USA) membranes. The membranes were blocked with Tris-buffered saline containing 0.3% Tween-20 (TBST) and 5% normal goat serum at 37°C for 60 min. Subsequent to blocking, the membranes were washed four times for 15 min with TBST at room temperature and then incubated with the following primary polyclonal antibodies: Rabbit anti-human EV71 (1:1,000; Millipore), rabbit anti-human CDK4, rabbit anti-human caspase-3, rabbit anti-human active caspase-3, rabbit anti-human Bcl-2, rabbit anti-human BAX and rabbit anti-GAPDH (1:1,000; all Cell Signaling Technology, Inc., Danvers, MA, USA). The membranes were washed four times for 15 min with TBST at room temperature. Following washing, the membranes were incubated at room temperature with peroxidase-linked goat anti-rabbit IgG secondary antibody (1:1,000; Santa Cruz Biotechnology, Inc., Santa Cruz, CA, USA) for 1 h. Protein bands were visualized by autoradiography, using an enhanced chemiluminescence kit (Pierce Biotechnology, Inc., Rockford, IL, USA).

### Flow cytometric (FCM) analysis of the cell cycle by propidium iodide (PI) staining

A total of 3×10^5^ cells per well were seeded in 6-well plates and cultured until 85% confluent. Each group of cells was washed with PBS three times, then collected by centrifugation (Allegra X-22R; Beckman Coulter, Miami, FL, USA) at 1,000 × g for 5 min. The cell pellets were subsequently resuspended in 1 ml PBS, fixed in 70% ice-cold ethanol and stored in a freezer for >48 h at −20°C. Prior to flow cytometric analysis, the fixed cells were centrifuged, washed twice with PBS and resuspended in PI staining solution (Sigma-Aldrich Chemie GmbH) containing 50 *μ*l/ml PI and 250 *μ*g/ml RNase A (Sigma-Aldrich Chemie GmbH). The cell suspension was incubated for 30 min at 4°C in the dark, and analyzed by FACS (FCM-500; Beckman Coulter). A total of 20,000 events were recorded for analysis using CellQuest™ software (BD Biosciences, Franklin Lakes, NJ, USA).

### MTT assay of cell proliferation

Each group of cells was seeded at 2×10^3^ cells per well in 96-well plates and cultured in DMEM supplemented with 10% FBS at 37°C with 5% CO_2_, until 85% confluent. MTT reagent (5 mg/ml; Sigma-Aldrich Chemie GmbH) was added to the cell medium at different time points and incubated at 37°C for an additional 4 h. The reaction was terminated by adding 150 *μ*l dimethylsulfoxide (Sigma-Aldrich Chemie GmbH) per well and the cells were lysed for 15 min, with the plates gently agitated every 5 min. The absorbance values were determined using an enzyme-linked immunosor-bent assay reader (Model 680; Bio-Rad, Hercules, CA, USA) at 490 nm.

### 2′-O-Me RNA transfected

The 2′-O-Me RNA oligonucleotide, targeting silenced miRNA let-7b, was synthesized by Shanghai GenePharma Co.,Ltd., (Shanghai, China). The SH-SY5Y cells were transfected with 20 *μ*M 2′-O-Me using Lipofectamine 2000 (Invitrogen Life Technologies), according to the manufacturer’s instructions.

### Statistical analysis

Each experiment was performed at least three times. The data are presented as the means ± standard error of the mean, where applicable, and the differences were evaluated using Student’s t-test with SPSS 18.0 statistical software (SPSS, Inc., Chicago, IL, USA). A P<0.05 was considered to indicate a statistically significant difference.

## Results

### EV71 induces SH-SY5Y human neuroblastoma cell apoptosis

**Figure 1:**
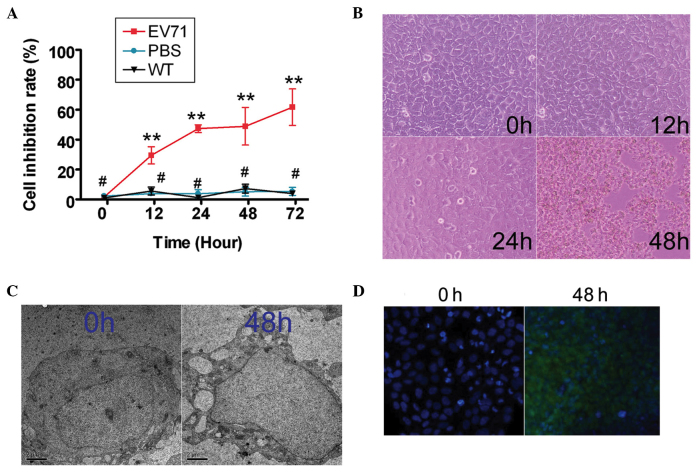
Enterovirus (EV)71 inhibited SH-SY5Y human neuroblastoma cell proliferation. (A) An MTT proliferation assay was used to determine the ability of EV71 to suppress SH-SY5Y cell proliferation at 0, 12, 24, 48 and 72 h after infection. Between 12 and 72 h, EV71-infected cells exhibited significant proliferation inhibition compared with control cells (^**^P<0.01 and ^#^P>0.05 vs. wild-type (WT) group; n=3). (B) Cell morphological analysis confirmed that 48 h after EV71 virus infection, SH-SY5Y cells exhibited signs typical of apoptosis (round, no longer adherent, fewer cell pseudopods). Magnification, ×200. (C) Transmission electron scanning analysis revealed EV71 virus particles within or surrounding SH-SY5Y cells (scale bar=1 *μ*m; magnification, ×10,000). (D) Terminal deoxynucleotidyl-transferase-mediated dUTP nick end labeling assay showed a strong fluorescein isothiocyanate hybridization signal in the EV71-infected group at 48 h, but not in non-infected SH-SY5Y cells (0 h; magnification, ×200).

To determine whether EV71 suppressed SH-SY5Y cell proliferation, an MTT assay was performed at 0, 12, 24, 48 and 72 h after infection (Fig. 1). At the 12–72 h time points, the wild-type (WT) and PBS groups were significantly less susceptible to the proliferation inhibitory effect of EV71 than the EV71 group (Table I). Furthermore, EV71 suppressed SH-SY5Y proliferation in a time-dependent manner, but no significant differences were identified between the WT and the PBS group at any time point. In addition, cell morphology confirmed that the original SH-SY5Y cells were healthy, and there was no indication of apoptosis or necrosis prior to EV71 infection (Fig. 1). However, 48 h after EV71 infection, SH-SY5Y cells exhibited features typical of apoptosis: The cells were round and no longer adherent, and fewer cell pseudopods were observed. In addition, TEM analysis revealed increased numbers of intracellular vacuoles in SH-SY5Y cells 48 h post EV71 infection, compared with the control cells at this time point. In addition, typical EV71 virus particles within or surrounding the SH-SY5Y cells were identified (Fig. 1). Furthermore, TUNEL assay revealed a strong FITC hybridization signal in the EV71 infection group at 48 h, that was not evident in the non-infected cells (Fig. 1). These results demonstrate that the EV71 virus induced SH-SY5Y apoptosis.

**Figure 2:**
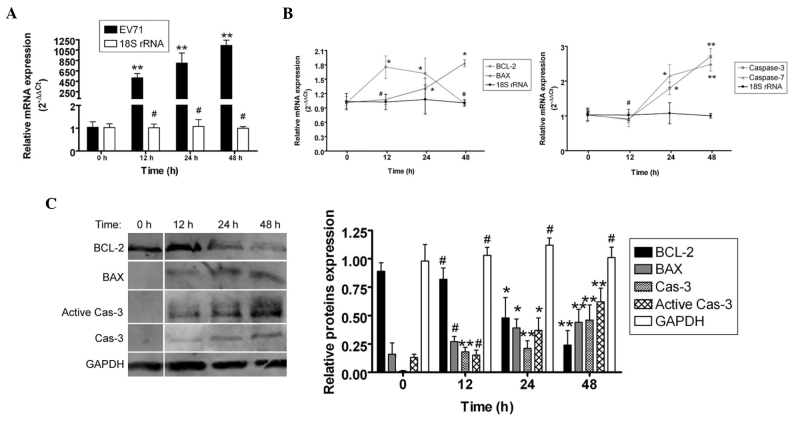
Enterovirus (EV)71-induced apoptosis-related protein expression. (A) The expression levels of EV71 core RNA in SH-SY5Y human neuroblastoma cells determined by reverse transcription polymerase chain reaction (RT-PCR), normalized to cellular 18S rRNA (^**^P<0.01 vs. 0 h; n=3). (B) RT-PCR revealed that mRNA expression levels of the apoptosis inhibitor Bcl-2 were markedly lower in the SH-SY5Y cells following EV71 infection between 12 and 48 h compared with non-infected cells (0 h). By contrast, mRNA expression levels of the apoptosis-promoting factors Bax, caspase-7 and caspase-3 were markedly higher in SH-SY5Y cells 12 to 48 h after EV71 infection compared with non-infected cells (0 h; ^**^P<0.01, ^*^P<0.05 and ^#^P>0.05 vs. 0 h; n=3). (C) Western blotting confirmed that Bcl-2 protein expression levels were significantly reduced in SH-SY5Y cells 12 to 48 h after EV71 infection compared with non-infected cells (0 h). Furthermore, Bax, caspase-3 and active caspase-3 protein expression levels were significantly elevated in SH-SY5Y cells 12 to 48 h after EV71 infection compared with non-infected cells. GAPDH served as a loading control (^**^P<0.01, ^*^P<0.05 and ^#^P>0.05 vs. 0 h; n=3).

To determine how EV71 virus induced apoptosis in the SH-SY5Y cells, qPCR and western blotting were used to measure the expression levels of apoptosis-related factors. qPCR revealed that the mRNA expression levels of the apoptosis inhibitor Bcl-2 were markedly lower in the SH-SY5Y cells 12–48 h post EV71 infection, compared with the non-infected cells (0 h). By contrast, the mRNA expression levels of the apoptosis-promoting factors Bax, caspase-7 and caspase-3 were markedly higher in SH-SY5Y cells 12–48 h post EV71 infection, compared with non-infected cells (0 h). In addition, western blot analysis found that the Bcl-2 protein expression levels were significantly reduced in SH-SY5Y cells 12–48 h post EV71 infection compared with non-infected cells (0 h). Furthermore, the Bax, caspase-3 and active caspase-3 protein expression levels were significantly elevated in SH-SY5Y cells 12–48 h post EV71 infection compared with non-infected cells (Fig. 2). These data indicate that EV71 virus stimulated the expression of apoptosis-related proteins to induce apoptosis.

### EV71 stimulates endogenous miRNA let-7b and inhibits CCND1 expression

**Figure 3:**
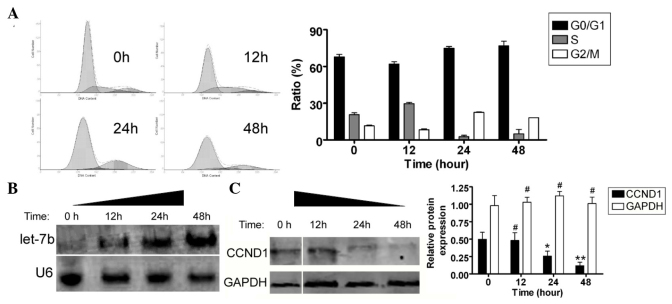
Enterovirus (EV)71 arrested the SH-SY5Y human neuroblastoma cell cycle via regulation of let-7b and cyclin D1 (CCND1) expression levels. (A) Flow cytometry results revealed that compared with non-infected cells, SH-SY5Y cells were arrested in G2/M phase of the cell cycle and the percentage of cells in the S phase was significantly reduced following infection. (B) Northern blot analysis indicated a marked let-7b hybridization signal in the EV71-infected group compared with the non-infected SH-SY5Y cells (0 h). (C) Western blotting confirmed that CCND1 protein expression levels were significantly reduced in EV71-infected SH-SY5Y cells at each time point compared with non-infected cells. GAPDH served as a loading control (^**^P<0.01, ^*^P<0.05 and ^#^P>0.05. 0 h; n=3).

FCM was used to determine whether EV71 influenced the SH-SY5Y cell cycle. Subsequent to co-culture with the EV71 virus, the SH-SY5Y cells underwent significant cell cycle arrest. Compared with non-infected cells, a greater number of SH-SY5Y cells were arrested in the G2/M phase and the percentage of cells in the S phase was significantly lower (Fig. 3A). These results suggest that EV71 significantly affected cell cycle regulation in the SH-SY5Y cells. Northern and western blotting were used to determine whether the expression levels of endogenous miRNA let-7b were different between EV71 virus-infected SH-SY5Y cells and control cells. The northern blot analysis revealed a marked let-7b hybridization signal in the EV71-infected group, compared with non-infected SH-SY5Y cells (Fig. 3B). Furthermore, western blotting confirmed that CCND1 protein expression levels were significantly reduced in the EV71-infected SH-SY5Y cells at each time point (0.481±0.192, 0.257±0.123 and 0.119±0.085, respectively), compared with the non-infected cells (0.496±0.178; Fig 3C). These data indicate that the expression levels of endogenous miRNA let-7b were significantly higher and those of CCND1 protein were significantly lower in the SH-SY5Y cells following EV71 infection.

### Inhibiting endogenous miRNA let-7b expression levels with 2′-O-Methyl-RNA maintains SH-SY5Y proliferation

**Figure 4:**
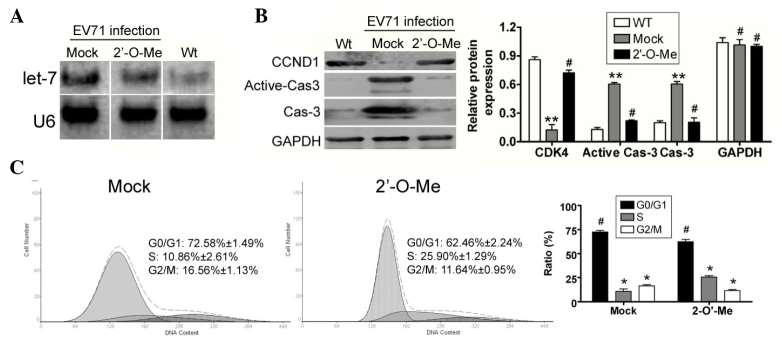
Inhibiting endogenous miRNA let-7b expression with 2′-O-Methyl-RNA maintains SH-SY5Y human neuroblastoma cell proliferation following enterovirus (EV)71 infection. (A) Northern blot analysis revealed a low let-7b hybridization signal in the 2′-O-Methyl-RNA-transfected group compared with the mock-transfected or wild-type (WT) groups. (B) Western blotting confirmed that cyclin D (CCND1) protein expression levels were significantly elevated following EV71 infection in 2′-O-Methyl-RNA-transfected SH-SY5Y cells. However, caspase-3 and active caspase-3 protein expression levels were significantly reduced following EV71 infection in 2′-O-Methyl-RNA-transfected SH-SY5Y cells. GAPDH served as a loading control (^**^P<0.01, ^*^P<0.05 and ^#^P>0.05 vs. WT; n=3). (C) Flow cytometry results revealed that in 2′-O-Methyl-RNA transfected SH-SY5Y cells, the cell cycle status improved and the percentage of cells in G2/M phase was significantly reduced (^*^P<0.05 and ^#^P>0.05 vs. mock-transfected group; n=3).

To confirm that EV71 induces host cell SH-SY5Y apoptosis by influencing let-7b, 2′-O-Methyl-RNA was used to inhibit endogenous let-7b expression levels. Northern blot analysis revealed significantly increased let-7b hybridization in the EV71-infected SH-SY5Y cells (mock group). However, a significant reduction in let-7b hybridization signal was observed in the EV71-infected 2′-O-Me group and in the SH-SY5Y cells without viral infection (WT group; Fig. 4A). In addition, western blot analysis confirmed that the protein expression levels of CCND1 were significantly increased in the WT and the 2′-O-Me groups, compared with the mock group (Fig. 4B). However, the protein expression levels of caspase-3 and active caspase-3 were significantly reduced in the WT and 2′-O-Me groups, compared with the mock group (Fig. 4B). In addition, compared with the mock group, the FCM results revealed that the cell cycle of the 2′-O-Me group was modified and the percentage of cells in G2/M phase was markedly reduced (Fig. 4C).

## Discussion

Thus far, miRNAs have been demonstrated to be important in the complicated interactions between virus and host in HFMD. The majority of reports indicate that miRNAs inhibit EV71 replication in host cells by downregulating the expression levels of viral core proteins (1,2,17,20–22). However, the present study was the first, to the best of our knowledge, to analyze the role of EV71 in stimulating the endogenous miRNAs of host cells in order to facilitate the induction of host cell apoptosis following infection. Typically, EV71 transfers genetic material into host cells through cell membrane receptors, then utilizes host cell machinery to assist viral processing (1,2), including replication and packaging, prior to producing viral particles (1,2). Simultaneously, host cells gradually undergo necrosis or apoptosis due to cell destruction by EV71. However, how the EV71 virus induces host cell apoptosis following infection remains unclear. Several studies have reported that host cellular miRNAs inhibit EV71 infection and replication, and that virus mutations escape suppression by cellular miRNAs (20–22). These findings suggest that since inhibition of viral replication and packaging of miRNAs occurs in host cells, certain miRNAs in host cells are assisting viral processing. The preliminary results of the present study suggest that when EV71 infected SH-SY5Y cells, the expression levels of endogenous, cellular let-7b were significantly increased. In addition, a number of studies have demonstrated that let-7b initiates cell cycle arrest and inhibits cell proliferation by targeting the expression of cell cycle-related proteins (23–26). Thus, as determined by these data, EV71 is hypothesized to inhibit host cell growth and promote apoptosis through stimulation of host let-7b expression.

In the present study, the SH-SY5Y cell line served as model host cells to determine injury following EV71 infection. EV71 infection was observed to undermine mitochondrial stability in these cells. Simultaneously, EV71 arrested cell cycle progression and subsequently inhibited the proliferation of host cells. EV71 stimulated the overexpression of apoptosis-related genes and induced host cell apoptosis. Conversely, through analysis of epigenetic regulation of EV71 in host cells, EV71 infection was found to stimulate endogenous miRNA let-7b expression; let-7b suppressed the expression of the target gene *CCND1* and induced normal cell cycle arrest in host cells. To further confirm that EV71 induced cell cycle arrest through let-7b, 2′-O-Methyl-RNA oligonucleotides were used to inhibit endogenous let-7b expression levels. The assay results revealed that following EV71 exposure, cell cycle arrest in the 2′-O-Methyl-RNA transfected group cell was significantly reduced compared with that in the mock-transfected cells. In conclusion, the present study demonstrates that EV71 inhibits growth and proliferation of host cells through stimulating the expression of miRNA let-7b. Furthermore, the findings suggest that miRNA let-7b is a potential candidate for antiviral therapy in HFMD.

## Figures and Tables

**Table I:** Analysis of SH-SY5Y cell proliferation inhibition rate following EV71 infection, by MTT assay.

Time (h)	EV71 (MOI=1; %)	PBS (%)	WT (%)
0	1.67±1.68	2.32±0.60	1.15±1.54
12	29.57±5.75**	3.90±1.54	5.65±2.64
24	47.36±2.52**	3.88±2.73	1.32±1.26
48	48.91±12.54**	5.22±2.90	7.27±2.98
72	61.68±12.19**	5.37±2.69	3.97±0.96

** P<0.05, vs. WT group; n=3. EV71, enterovirus 71; PBS, phosphate-buffered saline; WT, wild-type; MOI, multiplicity of infection.

## Abbreviations

HFMD: hand, foot and mouth disease
CVA16: Coxsackie virus A16
miRNA: microRNA
HMGA: H-RAS and high-mobility group AT-hook
